# Adaptive Structured Light with Scatter Correction for High-Precision Underwater 3D Measurements

**DOI:** 10.3390/s19051043

**Published:** 2019-03-01

**Authors:** Petter Risholm, Trine Kirkhus, Jens T. Thielemann, Jostein Thorstensen

**Affiliations:** Smart Sensor Systems, SINTEF Digital, Forskningsveien 1, 0314 Oslo, Norway; Trine.Kirkhus@sintef.no (T.K.); Jens.T.Thielemann@sintef.no (J.T.T.); jostein.thorstensen@sintef.no (J.T.)

**Keywords:** structured light, multi-frequency phase stepping, underwater, scatter correction, uncertainty

## Abstract

High-precision underwater 3D cameras are required to automate many of the traditional subsea inspection, maintenance and repair (IMR) operations. In this paper we introduce a novel multi-frequency phase stepping (structured light) method for high-precision 3D estimation even in turbid water. We introduce an adaptive phase-unwrapping procedure which uses the phase-uncertainty to determine the highest frequency that can be reliably unwrapped. Light scattering adversely affects the phase estimate. We propose to remove the effect of forward scatter with an unsharp filter and a model-based method to remove the backscatter effect. Tests in varying turbidity show that the scatter correction removes the adverse effect of scatter on the phase estimates. The adaptive frequency unwrapping with scatter correction results in images with higher accuracy and precision and less phase unwrap errors than the Gray-Code Phase Stepping (GCPS) approach.

## 1. Introduction

Traditional subsea inspection, maintenance and repair (IMR) operations are costly because they require manual control using Remotely Operated Vehicles (ROVs). One goal of the subsea industry is to move towards more autonomous IMR operations which are cost-effective and facilitates extraction of oil in more remote areas. To achieve robust and fault-safe autonomous IMR requires 3D sensors that can provide a high-resolution and -precision 3D view of objects of interest. Furthermore, if the 3D sensor can provide “error-bars” on the 3D data, the autonomous system can make more reliable decisions with regards to e.g., the likelihood that a pipeline is defect, how reliably a robotic arm can grip a handle, or determine whether a valve on the subsea template is open or closed.

Sonars have traditionally been the underwater 3D technology of choice because of its range and robustness against water turbidity. However, sonar’s image update rates are limited by the speed of sound, while diffraction limits the lateral resolution. The result is slow update rates and low-resolution 3D data which makes it impractical and imprecise to be used in many autonomous IMR operations.

Video cameras are extensively used on ROVs today as a guidance tool for the operators, as well as for some autonomy using e.g., markers [1]. Video cameras do not suffer from the same physical limitations as sonars. Cameras allow fast sampling rates and high lateral resolution, due to the high speed and short wavelengths of light. However, in practice the light-water interaction causes light attenuation and scattering which affects the performance of optical 3D systems. The effect of both attenuation and scattering are dependent on the turbidity of the water. Light attenuation limits the potential range of the system, while the scattering has primarily two effects. The backscatter causes a contrast degradation in the image while the forward scatter causes a blurring of features. Some typical approaches to remove the scattering effect on 2D images is to apply image de-hazing [2,3], de-convolution [4] or specific forward- or backward-scatter models [5,6].

Single camera systems are capable of extracting 3D information through photogrammetry [7]. Stereopsis is widely used as a 3D sensing technology, mainly because of its relatively simple and cheap construction [8]. A stereo-camera system consists of two calibrated cameras and a stereo-matching algorithm which searches for corresponding features along corresponding epipolar lines in the cameras [9]. Once a correspondence (disparity) is established, the distance can be estimated through simple triangulation. Establishing the feature correspondences in underwater imagery is challenging because of attenuation and scattering which reduces the image contrast, color separation and smears out image features [5]. Furthermore, many underwater structures are textureless which makes it almost impossible to find feature correspondences.

532 nm lasers are frequently used as a light source underwater to increase the imaging range by diminishing the effect of light attenuation and scattering. Triangulation systems project the laser light, typically as laser sheets [10,11,12] or points [13], and generally incorporate a relative slow scanning mechanism to cover an extended field of view. At short distances, the imaging sensor can easily be saturated because of the high energy focused light. An alternative faster approach is to illuminate the whole field-of-view with short powerful light pulses and use a fast range-gated camera to estimate the time-of-flight [14,15,16]. Range-gated solutions are effective solutions to suppress backscatter [15,17].

Structured light sensors typically use a DMD for fast time-multiplexing of spatially coded light [18]. The advantage over laser-based scanning approaches for subsea intervention and inspection is the potential for real-time frame-rates and high-resolution both laterally and depth-wise in the 0.5 m-2 m range. Typical patterns used are binary [18], Gray codes [19] or sinusoidal fringe patterns [20]. Gray-codes are often used to phase-unwrap the phase-maps from high-frequency phase-shifted sinusoidal patterns [21]. The depth-precision of such systems are limited by the number of codes that can be reliably segmented [22]. With increasing turbidity, the boundary between illuminated and non-illuminated areas are blurred and an accurate segmentation is difficult [23].

An alternative to using binary/Gray codes for phase unwrapping is to use multi-frequency phase-shifted (MFPS) projection and unwrap the phase based on the remainder theorem [24] or the phase uncertainty [25]. In this paper we propose an underwater structured light system which uses an MFPS method adapted for underwater use. Forward scattering of light in water can be viewed as a convolution with a symmetric point spread function (PSF). In an ideal setting, a symmetric PSF convolved with an infinite sinusoidal wave will not change the phase or frequency only the amplitude. Unfortunately, the projected sinusoid is bounded and height differences shifts the sinusoidal pattern such that it is not a spatial sinusoidal signal seen from the camera. Consequently, the scattering affects the phase estimate of sinusoidal fringe patterns. We propose models for removing the effect of both back- and forward-scatter. A relationship between the shot-noise of the signal and the phase-estimates is established. The phase uncertainty is used to unwrap the high-frequency phase estimates. The result is a dense depth map with corresponding depth uncertainties. We compare our results to GCPS.

## 2. Materials and Methods

In this section we first introduce the hardware that were used to produce the results in the paper. Even though the concepts of depth precision from phase stepping and phase unwrapping have been published elsewhere, we provide a short summary of them in Section 2.2 and Section 2.3 to introduce our notation. Section 2.4 and Section 2.5 summarizes our proposed methodology for adaptive phase unwrapping and scatter-removal.

### 2.1. Underwater Housing and Sensor

**Figure 1:**
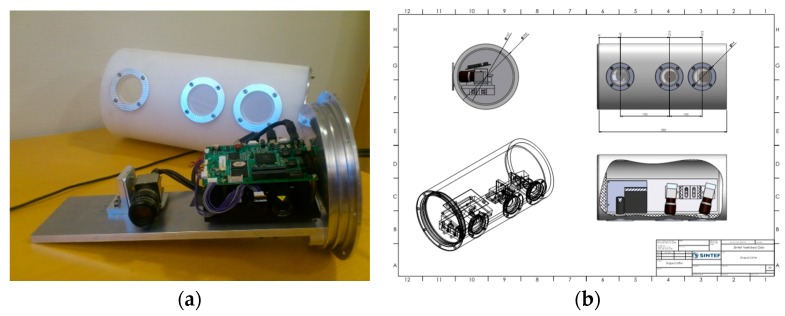
(**a**) Camera (right) and projector (left) mounted on an aluminium plate. Housing in the back. (**b**) Underwater housing overview and dimensions.

The structured light sensor is made up of a machine vision camera and a DMD (digital mirror device) projector (see Figure 1a). The projector is a LightCrafter RGB projector (DLP^®^ LightCrafter™ 4500, Texas Instruments, Dallas, TX, USA) which facilitates 120 Hz 8-bit pattern display. The machine vision camera is a BU238M USB 3.0 black and white camera (Toshiba, Tokyo, Japan) with a resolution of 1920 × 1200. The camera was operated with a gain of 4× which resulted in a digital number to photo-electron conversion factor γ=33. We only used the green LED of the projector to avoid chromatic aberration in the air-to-water transitions. The green part of the optical spectrum is also the least attenuated in water [5].

The underwater housing, which can be seen in Figure 1b, consists of a cylindrical plastic housing with windows for the camera and projector. The housing was fitted with three windows to facilitate adjusting the baseline. Longer baselines may be better for use-cases where long imaging distance is required, while shorter baselines are adequate for shorter imaging distances. The camera and projector are mounted on a metal plate which is attached to one of the end plates. The end plates are screwed onto the cylinder and sealed with a double set of O-rings. A 10-m hose is attached to the housing. We are currently using a USB 3.0 camera which does not facilitate longer cables than 10 m, however, by switching to a gigabit Ethernet camera we could potentially extend the cabling to 100 m if the use-case warrants it.

### 2.2. Depth-Precision of Phase Stepping

The phase stepping depth estimation procedure consists of capturing three or more images of phase-shifted sinusoids that are projected onto a scene and measuring the per-pixel phase shift. In this paper we focus on the case of four phase-shifted sinusoids where the unwrapped phase-shift can be estimated through [21]:

(1) $$\phi_{w}\left(x,y\right)=\arctan\left(\frac{I_{1}\left(x,y\right)-I_{3}\left(x,y\right)}{I_{2}\left(x,y\right)-I_{4}\left(x,y\right)}\right)$$

The projected sinusoids imaged in $I_{1},\;\ldots ,\;I_{4}$ are phase shifted with 90° with respect to each other. The images are represented by a signal level (amplitude) $I_{a}\left(x,y\right)=\sqrt{{\left(I_{1}\left(x,y\right)-I_{3}\left(x,y\right)\right)}^{2}+{\left(I_{2}\left(x,y\right)-I_{4}\left(x,y\right)\right)}^{2}}$ and a background signal level of $I_{b}\left(x,y\right)=\;\frac{1}{4}\overset{4}{\underset{i=1}{\sum }}I_{i}\left(x,y\right)-I_{a}\left(x,y\right)/2$ measured in units of photo-electrons. The total signal is determined as $I_{s}=I_{a}+I_{b}$. The phase uncertainty is dependent on the image noise, primarily shot-noise of each of the phase-shifted images, and can be shown to be [22]:

(2) $$\sigma_{\phi_{w}}\left(x,y\right)=\;\frac{\sqrt{I_{a}\left(x,y\right)+2I_{b}\left(x,y\right)}}{I_{a}\left(x,y\right)}$$

### 2.3. Phase Unwrapping

With sinusoids with more than one period, the phase estimate in (1) will be wrapped at 2π. To produce an unwrapped phase estimate:

(3) $$\phi \left(x,y\right)=\frac{\phi_{w}\left(x,y\right)+2\pi P\left(x,y\right)}{N_{p}}$$

we need to find P(x,y) which determines the sinusoidal period a pixel belongs to out of the $N_{p}$ periods that are projected. The pixelwise standard deviation of the unwrapped phase is:

(4) $$\sigma_{\phi }\left(x,y\right)=\frac{\sigma_{\phi_{w}}\left(x,y\right)}{N_{p}}$$

**Figure 2:**
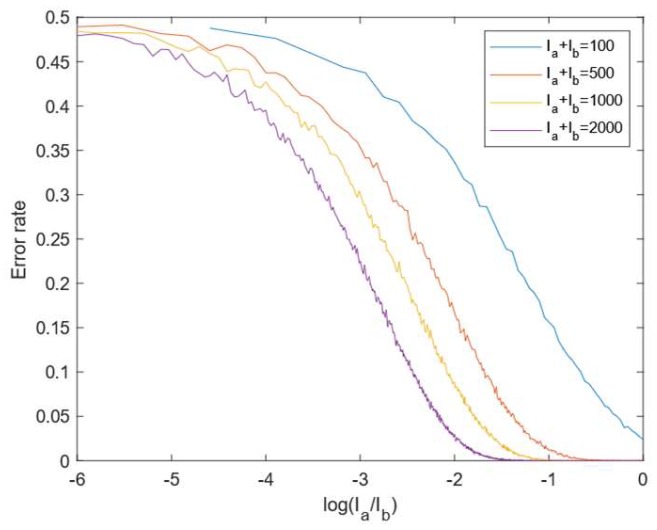
Gray code segmentation error rate for different signal levels (measured in photoelectrons).

A standard approach for estimating P(x,y) for patterns with more than a single frequency, is to use Gray codes [21]. To estimate an n-bit Gray-code, n+2 images are projected, viz. $I_{s},\;I_{b}$ and $I_{G_{1}},\;\ldots ,\;I_{G_{n}}$. The Gray codes are estimated based on $G_{i}=\frac{1}{2}I_{a}+I_{b}<I_{G_{i}}$. With shot-noise being the primary noise contribution, the noise of the black and signal pixels are $\sigma_{b}=\sqrt{I_{b}}$ and $\sigma_{s}=\sqrt{I_{a}+I_{b}}$. In Figure 2 we show the results of simulating the Gray code segmentation under different signal levels. The error rate varies with the total intensity level $I_{s}$ and the distribution of intensity between the background and signal. Most scenes have objects with varying albedos and consequently varying signal levels $I_{a}$ and $I_{b}$. The result is that in some areas of the scene, the signal level will be low, and the error rate of the Gray-decoding will be high which will result in pixels with wrong phase-unwrapping. This is particularly a problem underwater where the transition will be smeared out spatially because of forward scatter, i.e., $I_{a}$ will be lower around the transition than further away, and $I_{b}$ may be elevated because of backscatter. The Gray code approach to phase-unwrapping do not take the signal levels nor the signal uncertainty into account when choosing the appropriate period for a pixel which makes it an un-flexible approach.

### 2.4. Adaptive Multi-Frequency Phase Stepping (MFPS)

In this section we introduce a flexible approach for phase unwrapping which is data-driven. Phase-stepping provides higher depth-precision with higher frequency sinusoidal patterns according to Equation (4). However, the main problem is to accurately phase-unwrap the signal under different signal levels. The proposed approach uses the pixel-wise signal levels and the associated signal uncertainty to determine the highest sinusoidal frequency that can be phase unwrapped without ambiguity.

Assume we have projected a one-period sinusoidal pattern $\phi^{1}$ and an *n*-period sinusoidal pattern $\phi_{w}^{n}$. The traditional (MFPS) approach to phase unwrap $\phi_{w}^{n}$ is to solve for:

(5) $$P\left(x,y\right)=argmin_{i\in \left[0,\;\ldots ,\;n-1\right]}\left|\phi_{w}^{n}\left(x,y\right)+2\pi i-n\phi^{1}\left(x,y\right)\right|$$

Higher *n* will lead to higher error-rates when performing the phase unwrapping procedure because of the uncertainty in $\phi_{w}^{n}\left(x,y\right)$. We propose a data-driven approach to the MFPS unwrap procedure which based on the signal uncertainty decides how high an *n* a pixel can jump to.

The maximum number of periods that we can phase unwrap for a pixel from the base-frequency with an error rate less than $\gamma \sigma_{\phi }$, where γ is a parameter, is determined by the jump factor:

(6) F=⌊2πγσϕ⌋

Because this is a pixel-wise factor which varies from scene to scene, it is not practical to change the frequency of the projected patterns on-the-fly. In practice, a pre-defined schedule of sinusoidal frequency schedule is projected, and the pixels are individually phase-unwrapped to the highest permissible frequency according to the pixel-wise jump factor. Notice that if a pixel can jump with a factor of 2 from the base period, it can also jump with a factor of 2 from any higher periods that have been phase-unwrapped.

Determining a frequency schedule is a trade-off between the frame rate (with more frequencies, a greater number of patterns need to be projected which takes more time), the potential accuracy (the higher the frequency, the higher potential accuracy can be achieved), and the chance of falsely unwrapping the phase increases with larger frequency jumps. In this paper we propose to use a frequency schedule as follows: 1, 8, and 64. This results in 12 projected patterns which is the same required for the Gray code phase stepping (GCPS) approach for a 64 frequency signal (4 phase shifted 64-frequency sinusoids, one fully illuminated and on non-illuminated image, and 6 images to code the Gray code). Given that the jump factor is identical (a factor of 8) between the frequencies, we maximize the likelihood that as many pixels as possible will be able to jump to 64 as well as minimize the likelihood of phase unwraps. The advantage in comparison to Gray codes is that the phase estimates are adaptive with regards to the signal level. The phase estimates will have a predictable lower-bound phase-precision (uncertainty) with a low error rate with regards to the phase unwrapping independent of signal level. The phase-precision is a lower-bound because it considers shot-noise as the sole noise contribution, and disregards other noise sources such as from the turbidity.

### 2.5. Scatter Removal

The light scattering in turbid media generally affect imaging systems with active illumination in two ways. The backward scattering of the light increases the background level while the forward scatter blur image features. In this section we characterize the effect of these two on the projected images, and we propose algorithms to remove the scatter effect before applying the MFPS algorithm. We propose to remove backscatter by subtracting a turbidity dependent backscatter model from the raw images, while forward scatter is removed with an un-sharp filter. The effect of scatter removal on the phase estimates is shown in the results section.

#### 2.5.1. Backward Scatter Removal

The backscatter profile is a complex volumetric function which depends on the illumination volume, the water attenuation, and angular dependent scattering cross-section of the water. In MFPS, the illumination volume changes for each consecutive sinusoidal pattern that is projected, which makes this a difficult function to approximate through a parametric function. Instead, we sample the backscatter profile for each MFPS pattern at different turbidities by imaging into a void. This was performed in an aquarium (125 cm × 50 cm × 50 cm) where the turbidity was changed by adding clay to the water. The inside walls of the aquarium were covered in black fabric to make sure that all returned light was originating from backscatter. The turbidity, in terms of attenuation length, was measured as described in Appendix B.

**Figure 3:**
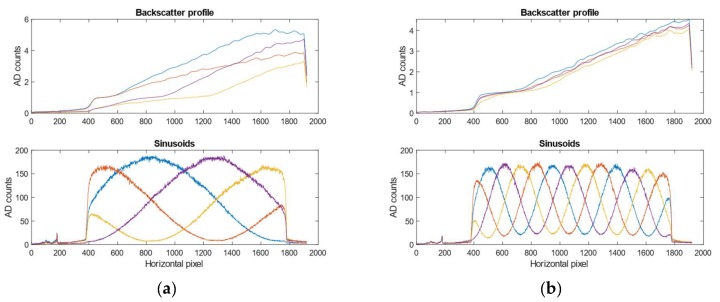
Backscatter profiles at turbidity with an attenuation length of $\lambda_{2}=1.1\;m$. (**a**) 1-period pattern. (**b**) 4-period pattern. The lower plots show the projected sinusoidal signal, while the upper plots show the backscatter profile. The colors correspond across sinusoids and the backscatter profile, e.g., the blue sinusoid corresponds to the blue backscatter profile. Notice how the profiles are dependent on the illumination profile for low frequencies. At higher frequencies, the illumination profile is more uniform and the backscatter profile for each is more constant across the shifted signals.

Figure 3 shows the backscatter profiles for the different shifted sinusoids at high turbidity for a 1 and 4 period sinusoidal pattern. The backscatter for a pixel changes with respect to the frequency *f* of the projected sinusoid, the phase shift *p* (1 through 4) of the sinusoid and the turbidity. Assuming we know the frequencies and the phase shifts that will be used, we create a backscatter model by projecting and imaging sinusoids with the mentioned frequencies and phases at pre-determined turbidities. Based on the sampled backscatter images we construct a 3-dimensional backscatter volume $B_{p}^{f}\left(x,y,t\right)$, where the two first dimensions define the image pixels, while the third dimension parametrizes the turbidity. The backscatter model is interpolated along the turbidity dimension to facilitate arbitrary turbidities within the original sampled turbidity range. In this paper we sampled the backscatter at four turbidities/attenuation lengths—viz. 0.8 m, 1.1 m, 2.0 m, 5.8 m. Assuming we are projecting a sinusoid with phase *p* and frequency *f*, at turbidity *t,* and the raw sampled image including backscatter is $I_{p}^{f}\left(x,y\right).$ The backscatter is simply subtracted from the raw image as follows: ${I^{\prime }}_{p}^{f}\left(x,y\right)=\;I_{p}^{f}\left(x,y\right)-B_{p}^{f}\left(x,y,t\right)$. This model assumes the backscatter is the same independent of the distance to objects in the scene.

#### 2.5.2. Forward Scatter Removal

**Figure 4:**
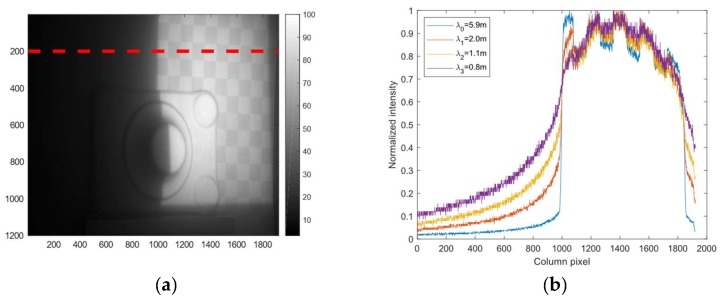
Point spread function of forward scatter. (**a**) Shows a projected Gray code. (**b**) Shows the normalized intensity profile along the red stapled line across turbidities. Notice the increase in width of the PSF with increasing turbidity. In the absence of forward scatter, the intensity left of pixel 1000 should have been zero.

Forward scattering causes a blurring of features. Figure 4 shows the capture range of the forward scatter PSF―it is increasing with turbidity and the pixels on the boundary of the image are even affected at high turbidities.

The width of the PSF is also dependent on the distance to the scene. The convolution between a perfect sinusoid and a symmetric kernel will not change the phase or frequency of the signal, only the amplitude. However, because the PSF is so wide, the boundaries of the pattern will affect the sinusoid far from the boundary and change the phase of it. Furthermore, after the sinusoid has reflected off a scene with depth differences, the reflected pattern will not be a spatial sinusoid because of the phase-shifts and the PSF will consequently change the phase/frequency of the pattern.

One approach for forward scatter removal is to model the PSF and use a deconvolution algorithm to remove the forward scatter effect. It is not obvious from Figure 4 which parametric function can accurately approximate the PSF. Furthermore, the PSF may vary somewhat over the image due to small changes in turbidity and the images may be noisy due to the turbidity and shot noise. All these factors make it difficult to design a reliable deconvolution algorithm. Instead we propose to use an unsharp filtering approach which is more robust to wide and spatially varying PSF and noise.

The unsharp filtering approach subtracts a low-pass filtered version of the image from the original image to enhance the high-frequency content:

$${I^{*}}_{p}^{f}\left(x,y\right)=\rho ({I^{\prime }}_{f}^{f}\left(x,y\right)-\theta \left(I_{f,p}\times k\left(w,\sigma_{I}\right)\right)$$

**Figure 5:**
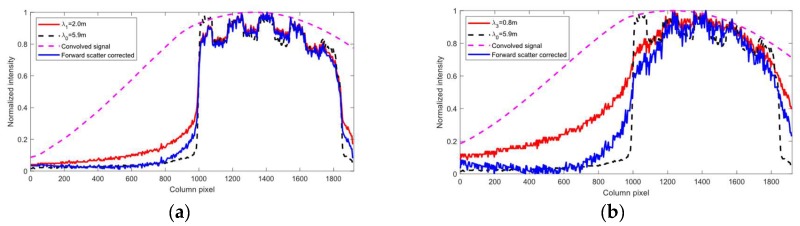
Forward scatter adjustment by unsharp filtering of the signal shown in Figure 4. (**a**) Turbidity $\lambda_{1}=2.0\;m$, $\theta_{t}=0.25,\;\rho_{t}=1.$ (**b**) Turbidity $\lambda_{3}=0.8\;m$, $\theta_{t}=0.6,\;\rho_{t}=1.$

The kernel *k* is a low-pass gaussian defined by the width in pixels *w* and the standard deviation $\sigma_{I}$. The subtraction scale $\theta_{t}$ and readjustment scale $\rho_{t}$ are turbidity dependent. Figure 5 shows the result from forward scatter adjustment using the unsharp filtering technique described here on the signal shown in Figure 4. The method reduces the effect of the PSF towards the left boundary of the image, while preserving the intensity features seen between pixels 1000 and 1800. In this paper we manually tune the scaling factors. However, they can be automatically estimated by finding the highest scaling factor which does not result in any negative valued pixels when applying the algorithm to the one-period sinusoidal patterns.

## 3. Results

All test data were acquired in an aquarium under controlled turbidity conditions. In this section we summarise the test setup and data acquisition followed by an evaluation of the performance of the scatter correction and compare the adaptive MFPS in relation to the GCPS algorithm. The conversion from phase to distance is performed with the simple calibration procedure outlined in Appendix A using L = 80 cm, B = 15 cm and a proportionality constant of 6.5.

### 3.1. Test Setup and Data Acquisition

**Figure 6:**
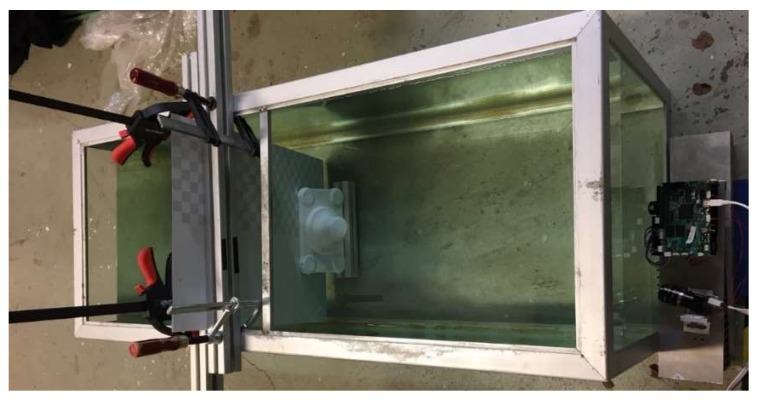
Test setup. The camera is placed on the right outside the aquarium of dimensions (125 cm × 50 cm × 50 cm).

An aquarium of dimensions 125 cm × 50 cm × 50 cm was used for the experiments and can be seen in Figure 6. The camera is placed outside the aquarium. The aquarium was filled with tap water. First, we acquired data of the white matte plate which was placed at 80 cm from the camera inside the aquarium and used it as a reference plane in the calibration procedure outlined in Appendix A. After acquisition of the reference plane, we changed it to the plate shown in Figure 6 where half the plate is matte white and the other half is covered with a checkerboard pattern (dark squares have albedo 0.5). This was to test the effect different albedos would have on the algorithm. Next, an object with a height of 11 cm was placed in front of the plate. We acquired data of this scene at clear tap water ($\lambda_{0}=5.9\;m$) and at three other turbidity levels ($\lambda_{1}=2.0\;m,\;\lambda_{2}=1.1\;m,\;\lambda_{3}=0.8\;m)$. Brown clay was added to the water successively to increase the turbidity. The turbidity was measured with the method described in Appendix B. At each turbidity level we acquired the following data:

(1) *GCPS data.* We used 6 Gray code patterns, one black and one white picture and 4 phase shifted 64-period sinusoids for a total of 12 images.
(2) *Adaptive MFPS data*. We used 4 phase-shifted sinusoids with 1, 8 and 64 frequencies for a total of 12 images.

**Figure 7:**
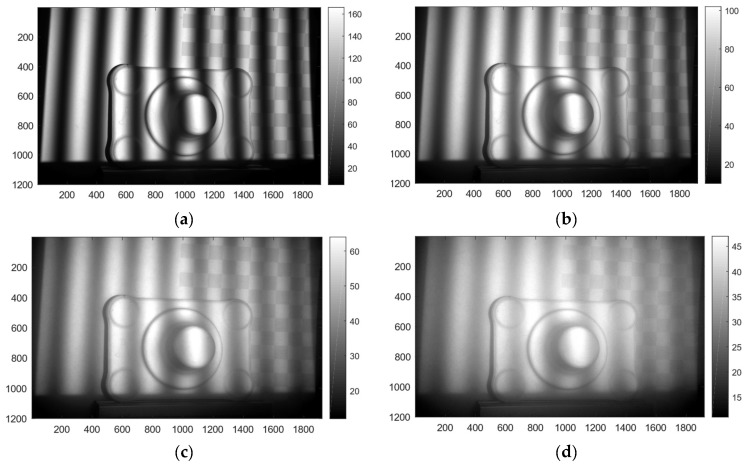
Images from the dataset of a sinusoid with eight periods at different turbidity levels. (**a**) Turbidity level $\lambda_{0}=5.9\;m$. (**b**) Turbidity level $\lambda_{1}=2.0\;m$. (**c**) Turbidity level $\lambda_{2}=1.1\;m$. (**d**) Turbidity level $\lambda_{3}=0.8\;m$.

Figure 7 shows images of the acquired datasets. The images show a sinusoid with frequency eight at different turbidity levels.

### 3.2. Effect of Scattering on Phase Estimates

**Figure 8:**
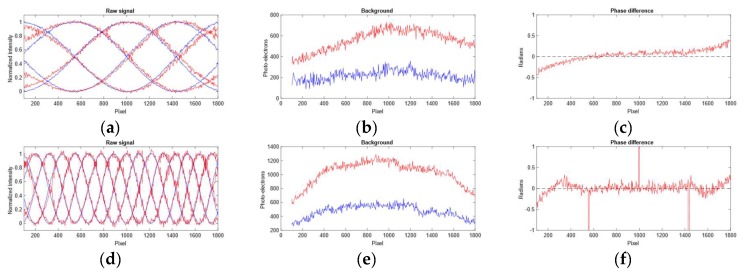
Effect of turbidity on phase estimates. The intensity and phase profiles are taken along row 200 of the images in Figure 7. Blue curves denote data from turbidity level $\lambda_{0}=5.9\;m$ while red curve denotes data from turbidity level $\lambda_{2}=1.1\;m.$ The upper and lower rows contain results for a sinusoid pattern with one and four frequency data respectively. (**a**,**d**) Normalized intensity plots. (**b**,**e**) Estimated background levels according to $I_{b}$. (**c**,**f**) Phase difference between the phase estimated at $\lambda_{0}$ and $\lambda_{2}$. The outliers in (**f**) occur at the phase wrapping boundary.

The scattering of light in water affects the phase estimates. In Figure 8 we show the combined effect of forward and backward scattering on the phase estimates. The phase deviation is much larger for the one frequency component than the eight frequency component. The scatter PSF has a wide reach and the boundary affects the sinusoid far towards the middle of the image as shown in Figure 8a. The effect is not as evident in Figure 8d because the boundary effect is more quickly mitigated because of the higher frequency signal. The increase in background signal ($I_{b})$ left to right shown in Figure 8b,e indicates that the backscatter is more prominent for pixels on the middle/right than for pixels on the left. This can be explained by the angular dependent cross-section of camera versus projector rays.

### 3.3. Backscatter Compensation

**Figure 9:**
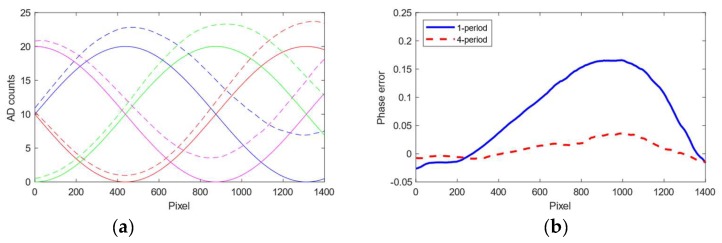
Effect of backscatter on phase estimates. (**a**) An ideal 1-period sinusoid is shown in solid, while the resulting curve when the backscatter profile from high turbidity is added to the signal is shown with the stapled curve. (**b**) The phase error caused by backscatter in high turbidities. We have included the phase error for the 1-period pattern and the 4-period pattern. The error is more prominent for the low period patterns.

As shown in Figure 3, backscatter affects the sinusoidal components differently. In Figure 9 we add the estimated backscatter contributions to ideal phase-shifted sinusoids for increasing turbidities and estimate the phase difference in relation to the ground truth. The maximum phase deviation at the highest turbidity reaches 0.2 radians which we can adjust for with the backscatter compensation. The effect is not as prominent for higher period patterns because the integral of the illumination profile along camera rays in this case will cross many periods and the backscattered signal over the image will even out. Using the simplified calibration formula in Equation (A1) with a baseline of 15 cm and a distance to the target of 80 cm and a proportionality constant of 6.5, a 0.2 radian error equates to a metric error of approximately 6.9 cm. Correcting for this bias caused by the backscatter improves the accuracy for the low SNR pixels that are not able to jump to high-frequency sinusoids. The bias is most prominent for the low frequency sinusoids, which means that the phase is shifted in relation to the high-frequency sinusoids. If this effect was uncorrected, at extreme cases there may occur a systematic error in the phase unwrapping when jumping from low frequencies to high frequencies.

**Figure 10:**
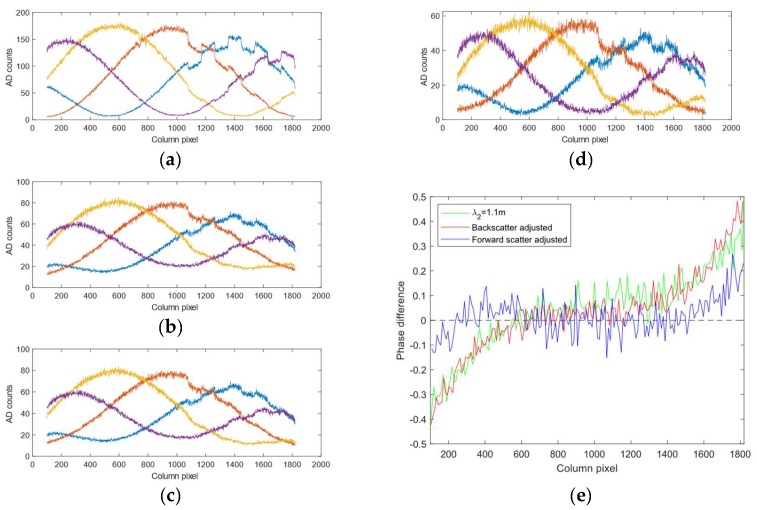
Scatter compensation at turbidity level $\lambda_{2}=1.1\;m.$ The intensity and phase profiles are along the row 200 of the images in Figure 7. (**a**) The raw signal the sinusoidal components with one frequency at turbidity level $\lambda_{0}=5.9\;m.$ (**b**) The raw signal of the sinusoidal components with one frequency at turbidity level $\lambda_{2}=1.1\;m.$ (**c**) The sinusoidal components after backscatter correction. (**d**) The sinusoidal components after backscatter and forward scatter correction. (**e**) The phase difference between the raw phase and scatter corrected phase at $\lambda_{2}$ versus the phase at $\lambda_{0}$.

In Figure 10 we show results after backscatter correction at a turbidity of $\lambda_{3}=0.8\;m$ on the dataset in Figure 7. We only show the effect for the one-period pattern because that is where the backscatter effect is strongest as shown in Figure 3. The effect on phase is in line with the simulated results shown in Figure 9, i.e., the backscatter corrects for phase differences to the right in the picture.

### 3.4. Forward Scatter Compensation

Figure 10 shows the effect of forward scatter removal on the backscatter removed intensity profiles along row 200 of the dataset in Figure 7 at $\lambda_{2}=1.1\;m$. We used scale factors of θ=0.5 and ρ=1.4, and a Gaussian low-pass filter with w = 1000 and $\sigma_{I}=800$. One can see that the components crossing (e.g., the location where the purple and orange curve crosses at around pixel 300) after scatter correction in (d) are more in line with the component crossing in (a) than what they are in (b). The effect on the phase estimates can be seen in (e) where the deviation from the phase at $\lambda_{0}$ is largely corrected for, except for at the boundaries.

### 3.5. MFPS versus GPCS

**Figure 11:**
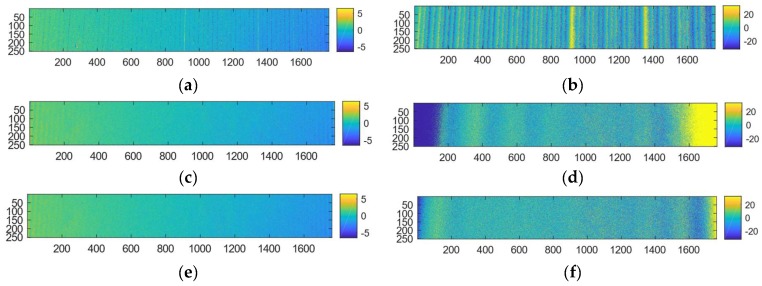
MFPS versus GCPS. We show the distance measurements on an area of 250 × 1800 of the top flat part of the images in Figure 7. The colorbars denote mm. Top to bottom rows contains results from GCPS, MFPS and the scatter corrected MFPS, respectively. The left and right column contains results from turbidities $\lambda_{1}=2.0\;m$ and $\lambda_{3}\;$= 0.8 m, respectively.

In Figure 11 we show comparative results between GCPS and MFPS with scatter correction. Even in relatively clear water ($\lambda_{1})$ the phase unwrapping of the GCPS produces errors. This is evident from the errors along vertical parallel stripes in the GCPS phase images. Notice that while turbidity increases the errors along the Gray code boundaries for the GCPS method, increasing turbidity increases the errors on the boundary of the plate for the MFPS which is not scatter corrected. This is in line with the scatter effect that was shown in Figure 10. Much of the boundary effect is removed with the proposed scatter correction algorithm.

In Table 1 we show qualitative results based on the areas shown in Figure 11. The error estimates are taken as the percentage of pixels in the full region shown in Figure 11 that have a distance from the ground truth which is more than $\pm \frac{2\pi }{64}$. This would indicate the number of pixels that have been wrongly phase unwrapped. The error, i.e., the accuracy and precision of the error, is estimated over a 100 × 100 area in the middle of the region shown in Figure 11. The last column denotes the predicted phase standard deviation using Equation (4). The results show that the predicted phase is a lower bound for the MFPS algorithm, mainly because it only considers shot noise. It is a better predictor at low turbidity because the particles causing turbidity adds considerable noise to the individual phase images. The scatter corrected MFPS algorithm contains less errors and provides a significant increase in precision and accuracy across the turbidities.

The MFPS algorithm unwraps the phase in multiple stages. In this paper we have used the frequency schedule 1, 8 and 64. In Table 2 we show the quantitative results for each of these phases at turbidity $\lambda_{2}=1.1\;m$**.** These results indicate the performance that can be achieved by e.g., only using the 1 or 8 frequencies. We found that the phase uncertainty allowed all pixels perform a frequency jump of 8. At low turbidity, the signal allowed most pixels to jump directly from the base frequency to 64.

**Figure 12:**
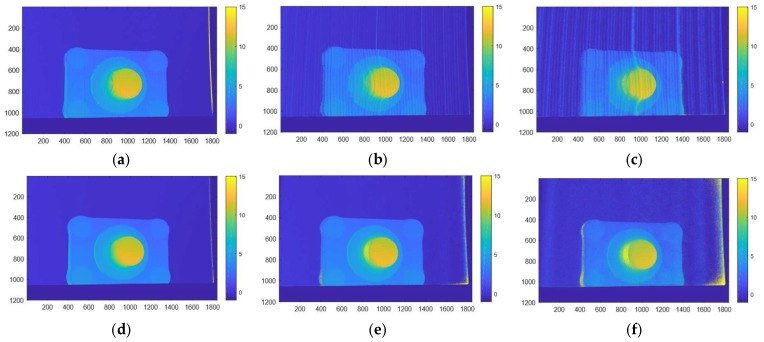
Qualitative results from imaging the object in Figure 7. Results in cm. (**a**–**c**): Results from GCPS. (**d**–**f**): Results from scatter corrected MFPS. The three columns represent turbidities (left to right) $\lambda_{0}=5.9\;m$, $\lambda_{2}=1.1\;m$ and $\lambda_{3}=0.8\;m$.

In Figure 12 we show qualitative results from imaging the object in Figure 7. We compare the proposed scatter corrected MFPS results with the results using GCPS. These images support the quantitative results: the scatter corrected MFPS has significant fewer outliers, higher accuracy and precision and degrade better with increasing turbidity.

## 4. Discussion

We have introduced a structured light method for high-precision depth measurements in turbid water. The method is based on the MFPS phase unwrapping method but includes a predictable lower-bound on the phase/distance uncertainty which is used to determine the highest reliable frequency that can be unwrapped. Furthermore, the effect of scattering has been investigated and methods that reduce the scatter effect on the phase estimates have been proposed. The results show that the method provides more accurate and precise depth estimates, and degrades better and more predictable with turbidity, than GCPS.

For the examples provided here, the pixel-wise uncertainty was low enough to be able to jump according to the frequency schedule that was used, i.e., 1, 8, 64. However, we still observe that pixels are wrongly unwrapped. This can be explained by several factors. First, the uncertainty is only a lower-bound because it only relies on the shot noise. The turbidity is the major factor, besides shot noise, that contributes to the increase in phase uncertainty. With increasing turbidity, more particles are present and moving in the water such that the pixel-wise scatter effect may be different from exposure to exposure and thereby adding noise to the measurements. However, this effect is difficult to parametrise and robust ways of quantifying this noise will be part of future work. If the shot-noise is the limiting factor to achieve a frequency jump, one solution to reduce the noise is to adaptively bin pixels. If we reduce the shot-noise by a factor of three by binning 3 × 3 pixels, the frequency jumping factor will increase by the same factor.

In this paper we used three frequencies and four phase shifted sinusoids for each frequency to perform the distance measurements. Consequently, 12 images were acquired to perform a distance measurement, which takes approximately 100 ms using the current system. In dynamic situations where the camera or scene is moving, this may be too long to avoid motion artefacts. One solution may be to use only three phase shifted sinusoids at the cost of higher phase uncertainty, or reduce the frequency schedule to only contain the base frequency and a higher frequency. The higher the frequency, the more accurate the distance estimate will be if the phase uncertainty permits to unwrap the signal. The minimum number of exposures that are required is 3 if only the base frequency is used, or 6 if another higher frequency is also used. If high precision estimates are required, and very reliable phase unwrapping, more frequencies can be used.

The backscatter primarily affects the lower frequencies and the rightmost pixels. This is because camera rays corresponding to pixels on the right cross the whole projector illumination volume before hitting a target. Consequently, the backscattered light will be different for the different shifted sinusoids for low-frequency signals. For higher-frequency signals, the effect cancels out because a camera ray crosses multiple peaks of the projected signal. The forward scatter is caused by a large PSF which is convolved with the projected signal. Boundary effects are the major contributor to the phase deviations we observe. The unsharp-filter can adjust for most of the forward scatter effect even at high turbidities.

We compared the proposed method with a standard GCPS method without error correction. It may be that using the error correction of the Gray codes could improve the GCPS results somewhat compared to the results reported here. We find that MFPS provides more accurate and precise results across turbidities and contains less phase unwrap errors due to the removal of the scattering effects. The results for both GCPS and MFPS are computed using the same number of patterns. While GCPS is fast and only rely on pixel-by-pixel computations, the scatter corrected MFPS is slower because it requires the application of a large convolving filter in the un-sharp filtering procedure and that the solution of Equation (5) is not as trivial as computing the Gray code. On a standard laptop running Matlab with non-optimized code, the Gray code method runs in 0.6 s, the standard MFPS runs in 2.6 s while the forward scatter removal filter adds another 9 s. However, we believe that a clever implementation on the GPU can reduce this gap considerably.

## Figures and Tables

**Table 1:** Errors in third column are defined as percentage of pixels with error larger than $\pm \frac{2\pi }{64}$ from the phase of the reference plane in the area shown in Figure 11. The accuracy and precision in the fourth column is defined as the mean and standard deviation (in millimeters) of the difference between the measured distance and the ground truth over a 100 × 100 area in the middle of the area shown in Figure 11. The predicted precision is computed using Equation (4).

Turbidity	Method	Errors #Wrongly Unwrapped/#Pixels	Accuracy/Precision Mean ± Std (mm)	Predicted Precision $\sigma_{\phi }$ (mm)
$\lambda_{0}=5.9\;m$	GCPS	0.8	−0.27±2.7	0.3
	MFPS	0.0	0.0±0.4	0.3
	Scatter MFPS	0.0	0.0±0.4	0.3
$\lambda_{1}=2.0\;m$	GCPS	1.4	−0.3±5.4	0.8
	MFPS	0.4	−0.2±1.0	0.8
	Scatter MFPS	0.3	−0.2±1.0	0.7
$\lambda_{2}=1.1\;m$	GCPS	1.1	0.8±9.2	2.2
	MFPS	7.1	0.2±3.9	2.2
	Scatter MFPS	0.5	0.3±4.1	1.4
$\lambda_{3}=0.8\;m$	GCPS	4.3	3.9±17.4	3.7
	MFPS	21.5	−2.9±13.1	3.6
	Scatter MFPS	5.4	−0.8±13.6	2.1

**Table 2:** Results from $\lambda_{2}=1.1\;m$. We show the results for the intermediate steps in the MFPS algorithm, i.e., the frequencies 1, 8 and 64. Description of the columns is given in Table 1.

Method	Errors (%) #Pixels/#Wrongly Unwrapped	Errors (mm) Mean ± Std	Predicted Error (mm) $\sigma_{\phi }$
GCPS	1.1	0.8±9.2	2.1
MFPS (1)	56.4	29.7±10.7	9.9
MFPS (8)	7.0	0.1±4.4	4.0
MFPS (64)	7.1	0.2±3.9	2.1
Scatter MFPS (1)	14.5	1.8±14.9	8.9
Scatter MFPS (8)	0.3	1.2±4.5	2.6
Scatter MFPS (64)	0.5	0.3±4.0	1.3

**Figure A1:**
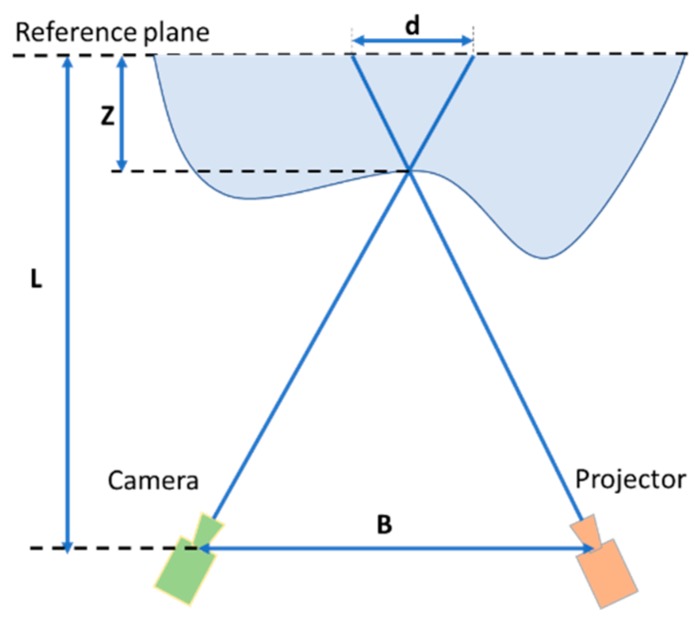
Overview of the calibration setup. The calibration uses the relative phase-shift between a reference plane and the object to compute the height Z.

## Appendix A. Calibration

Air-based structured light systems can typically be calibrated in essentially a similar way as traditional stereo-systems [26]. However, the validity of the pin-hole camera model in water is questionable because of the air-water refraction and more complex models are required to establish an accurate calibration model [27]. Since the focus of this paper is on the methodology for accurately estimating the phase-shift in scattering media, we use a simplified calibration methodology [28] which provides a metric distance map, but not a Euclidean point cloud. However, any calibration designed for underwater structured light systems can be used to convert the phase-maps to Euclidean point clouds.

Assume we have a camera-projector base line B and a distance *L* to a reference plane, as shown in Figure A1. Using simple trigonometry, we find the following relationships:

$\frac{Z}{L-Z}=\frac{d}{B}$, or $Z=\frac{L-Z}{B}d$ where *Z* is the height over the reference plane. Simplifying the relationship leads to:

(A1) $$Z\approx \frac{L}{B}d\;\propto \frac{L}{B}\left(\Phi -\Phi_{ref}\right)$$

where we exchange the distance shift *d* with the estimated phase-shift $\Phi -\Phi_{ref}$.

## Appendix B. Attenuation Measurements

The attenuation of light in water is determined by the attenuation coefficient *k* in $I_{z}=I_{0}e^{-z/\lambda }$, Where $I_{0}$ and $I_{z}$ are the intensity of a light source at distance 0 and *z*. It is difficult to measure $I_{0}$ directly, so instead we measure the log intensity at different distances and find the zero crossing which is an estimate of $I_{0}$. Consequently, by measuring $I_{z}$ at distance *z* for a new turbidity we can determine the attenuation coefficient by $\lambda =-\frac{z}{\ln\frac{I_{z}}{I_{0}}}$.
